# Knowledge, Attitudes, and Practices Related to Preoperative Chemoradiotherapy in Rectal Cancer Patients

**DOI:** 10.1155/2016/1081374

**Published:** 2016-09-27

**Authors:** Xingxing Chen, Ruifang Lin, Huifang Li, Meng Su, Wenyi Zhang, Xia Deng, Ping Zhang, Changlin Zou

**Affiliations:** Department of Chemoradiation Oncology, The First Affiliated Hospital of Wenzhou Medical University, Wenzhou, Zhejiang 325000, China

## Abstract

*Background*. The aim of this study is to assess the knowledge, attitudes, and practices related to pre-CRT in patients of stage II/III rectal cancer.* Materials and Methods*. Questionnaires regarding the knowledge, attitudes, and practices of pre-CRT were mailed to 145 rectal cancer patients in II/III stage between January 2012 and December 2014, and 111 agreed to participate and returned completed questionnaires to the researcher. Logistic regression model was used to compare sociodemographic characteristics, knowledge, and attitude with practice, respectively.* Results*. A total of 145 patients were approached for interview, of which 111 responded and 48.6% (54) had undergone pre-CRT. Only 31.5% of the participants knew that CRT is a treatment of rectal cancer and 39.6% were aware of the importance of CRT. However, the vast majority of participants (68.5%) expressed a positive attitude toward rectal cancer. Multivariate logistic regression analysis revealed that knowledge level (*p* = 0.006) and attitudes (*p* = 0.001) influence the actual practice significantly. Furthermore, age, gender, and income were potential predictors of practice (all *p* < 0.05).* Conclusion*. This study shows that, despite the fact that participants had suboptimal level of knowledge on rectal cancer, their attitude is favorable to pre-CRT. Strengthening the professional health knowledge and realizing the importance of attitudes may deepen patients' understanding of preoperative therapy.

## 1. Introduction

Rectal cancer is one of the most common cancers in the world after lung and prostate cancer among men and after breast and lung cancer among women, and it ranks as the major cause of cancer death in the Chinese compared with other races [1, 2]. Local recurrence and distant metastasis are major treatment failures of rectal cancer. Over the past two decades, the surgical techniques have improved dramatically such as total mesorectal excision (TME) reducing the local recurrence rates to <8% [3–5]. In Western countries, the addition of (neo)adjuvant therapy has led to improvements in post-TME local control [6]. Preoperative chemoradiotherapy (pre-CRT) can significantly enhance the pathological response in stage II/III rectal cancer as compared with surgery alone or surgery plus irradiation [7, 8].

A previous study has indicated that patients treated with pre-CRT had distinctly lower local failure rates and increased rate of sphincter preservation than those receiving postoperative chemoradiotherapy (post-CRT) [9]. Therefore, pre-CRT has now become the standard treatment for stage II and III disease. However, a large proportion of patients remain resistant to pre-CRT. One main barrier to preoperative therapy uptake is the knowledge related to help-seeking behavior and the choice of treatment schedule [10]. Previous studies have shown that an emphasis on knowledge of cancer can result in positive attitudes and practices clinically [11, 12]. However, there are few studies concerning the knowledge of rectal cancer in China. The aim of this study is to assess the knowledge, attitudes, and practices related to pre-CRT in rectal cancer patients.

## 2. Materials and Methods

### 2.1. Participants

All patients diagnosed with clinical stage II/III (American Joint Committee on Cancer version 6: pelvic magnetic resonance imaging (MRI) was used to define T category and N category) rectal cancer in the First Affiliated Hospital of Wenzhou Medical University between January 2012 and December 2014 were evaluated. Eligibility criteria included histopathologically confirmed, resectable adenocarcinoma with the inferior margin within 12 cm from the anal verge and agreement to participate in this clinical trial. Patients had adequate bone marrow and organ function and no previous chemotherapy or radiation for rectal cancer. Excluded patients were those whose contact information was unavailable or who were dying, had histories of rare tumor, such as melanoma and metastatic cancers, or had contraindications to chemoradiotherapy.

A total of 145 patients were eligible, among which 119 patients responded to the questionnaires. Review of finished questionnaires revealed that 8 had incomplete data. By comparing the remaining 111 patients with the 34 patients who did not respond or who returned incomplete data, we found that there were no significant differences in their sociodemographic characteristics and the rate of pre-CRT. Subsequent analysis was based on data obtained from 111 respondents who returned completed questionnaires (the effective response rate was 76.6%).

### 2.2. Questionnaire

A draft of the questionnaire was developed by a panel of experts including three professors of radiology and one colorectal surgeon and an epidemiologist with substantial experience in questionnaire studies. Then we conducted a presurvey among all rectal cancer inpatients in our hospital. Each controversial item was discussed at a conference and the preliminary questionnaire was formed based on these discussions. In order to further improve the feasibility of the questionnaire, we carried out a pilot test among the eligible patients in the outpatient clinic. They were asked to read the questionnaire and then undergo a semistructured interview addressing each question with respect to relevance, importance, and wording. The patients were also asked whether there was any other important component missing in the draft. The test was performed on five randomly selected patients. After the tests, the questionnaire was revised according to the feedback, and a second round of pilot test was performed on 10 patients following a similar procedure. Finally, the formal questionnaire was done.

The questionnaire was comprised of four sections to gather information about the sociodemographic characteristics of the participants, knowledge, attitude, and practice regarding rectal cancer and pre-CRT.

The sociodemographic characteristics included age, gender, educational background, occupation, marital status, and per capita family monthly income. The knowledge was assessed via a 6-point scale which had dichotomous response, that is, yes and no. Each positive response was scored as 1 and negative as 0. A score of 0–2 was considered as low knowledge level, 3-4 as middle level, and 5-6 as high level. Attitude was assessed by 5 statements regarding rectal cancer and pre-CRT responses to them were categorized as 2-point scale: yes or no. Attitude was considered as favorable to pre-CRT if four or more “yes” responses were gleaned. Those who received the pre-CRT were regarded as having good practice.

### 2.3. Data Analyses

Data analysis was conducted by using a statistical software program, SPSS version 19.0. Univariate logistic regression analyses were performed for sociodemographic characteristics, knowledge, and attitude, respectively, and multiple logistic regression analysis was used to identify the predictors of the positive practice (factors from univariate analysis with *p* < 0.05 were integrated into multivariate analysis). Stepwise entry of the variables used *p* value criteria of 0.05 and 0.10 for entry and removal. The *p* value of less than 0.05 was deemed as significant. *p* < 0.05 was considered to indicate statistical significance.

### 2.4. Ethics Approval

All procedures performed in the study involving human participants conformed to the ethical standards set by the Ethics Committee of the First Affiliated Hospital of Wenzhou Medical University, Wenzhou, China, and to the 1964 Helsinki Declaration and its later amendments or comparable ethical standards. Informed consent was given by all participants engaged in the study.

## 3. Results

Of 145 patients to whom the questionnaire was administered, 111 responded, resulting in a 76.6% response rate. 48.6% (54) of the patients received the pre-CRT among all the respondents and the rest (57) were against pre-CRT. The sociodemographic profiles of the participants are presented in Table 1. About two-thirds of respondents were male (66.7%) and over 55 years old (67.5%). Only a few patients had high school or higher level of education (4.5%), and 24.3% patients had no educational background at all. The majority of the participants were employed (64.9%).

Table 2 depicts the responses of research participants pertinent to knowledge and attitude and the distribution in practices. The knowledge regarding the chemoradiotherapy was limited as 31.5% of all the participants knew that CRT is a treatment of rectal cancer and 39.6% were aware of the importance of CRT. A slightly high level of knowledge about the advantages of preoperative chemoradiotherapy was shown (68.5%). Nevertheless, the knowledge levels of those who had previously received the pre-CRT (87.0%) were obviously higher than those who had not (49.1%). About one-fourth of patients thought that the pre-CRT will delay surgery timing (26.1%). The approaches to acquiring the knowledge of pre-CRT are shown in Table 3. 46.9% lacked the knowledge and those who were well informed in this regard basically obtained the knowledge from medical personnel (39.6%).

Despite the poor knowledge, most participants held a positive attitude toward rectal cancer (Table 2). The vast majority of participants were willing to consult doctor when getting sick (92.8%) and trusting the doctor (95.5%), and more than half were confident about the treatment (59.5%). Most families firmly supported and encouraged the patients to take the pre-CRT (87.4%).

Results of univariate logistic regression of sociodemographic characteristics, knowledge, and attitudes are presented through Tables 1 and 4, respectively. Univariate analysis showed that age, gender, education, income, knowledge level, and attitudes were significantly associated with the use of pre-CRT (all *p* < 0.05). Multivariate logistic regression analyses are shown in Table 5. Four sociodemographic factors (age, gender, education, and income), knowledge, and attitudes feature prominently in patients' practices (*p* < 0.05). Therefore, the high level of knowledge (OR 11.400; 95% CI 2.263–57.425) and favorable attitudes (OR 8.522; 95% CI 2.297–31.616) significantly resulted in the positive practice. Furthermore, age, gender, and income were potential predictors of practice.

## 4. Discussion

The combination of radiotherapy and chemotherapy has been shown to reduce local recurrences and to improve survival for locally advanced rectal cancer [13]. Compared with postoperative CRT, the preoperative approach was superior in terms of treatment compliance, toxicity, and sphincter preservation in patients [6, 9]. The purpose of this study is to evaluate whether knowledge and attitudes are the contributing factors in the choice of pre-CRT. The result shows suboptimal level of knowledge but positive attitude toward rectal cancer and pre-CRT. In our study, only 31.5% of all the participants knew that CRT is a treatment of rectal cancer and 39.6% were aware of the importance of CRT. The rate of patients with no pre-CRT (49.1%) was obviously lower than those with pre-CRT (87.0%) in the knowledge degree of the advantages of pre-CRT. Furthermore, only 30.7% grasped a high level of knowledge (Table 4). The similar results of poor knowledge of preoperative therapy were shown in breast cancer patients [14]. And there exists significant association between knowledge level of participants and the positive practice (*p* < 0.05) (Table 5). Therefore, strengthening the health knowledge for patients is necessary for increasing the rate of preoperative treatment.

Although highly educated patients generally participate more actively in medical consultations [15], there was no significant association between education level of patients and the acceptance of pre-CRT in this study (Table 4). A similar finding was reported in a Swedish study [16]. This result indicates that the general knowledge level had no influence on participants' practice. However, this conclusion needs to be validated by large-scale clinical trials.

When referring to the approaches of the knowledge of pre-CRT, the majority (39.6%) expressed that they were informed by consulting medical workers. Only 4.5% of the participants' acquired the knowledge from other patients who received radiotherapy previously, networks, and various kinds of media, respectively. The findings are similar to studies done by Hammick [17]. Hence, medical staff ought to actively promote the role of preoperative therapy, explain the effect and advantages of pre-CRT at full steam, and improve the patients' professional knowledge as far as possible. For instance, hospital can arrange for patients relevant lectures, set up the health knowledge billboard, and increase the communication between patients and doctors.

When it comes to the attitude toward rectal cancer and its treatment, most respondents stayed positive. In this study, the vast majority of participants were willing to consult doctor when getting sick (92.8%) and trusting the doctor (95.5%). More than half were confident about the treatment (59.5%) and 87.4% get firm support and encouragement from their family members. Besides, the favorable attitudes, to a large extent, resulted in the positive practice (*p* = 0.001) (Table 5). There were few published articles on the relationship between patients' subjective factor and the selection of therapeutic schedule so far. Medical workers are supposed to realize the importance of attitude, strengthen psychological support, and enhance their confidence in fighting against cancer.

Further association was found between sociodemographic characteristics and practice. On multivariate logistic regression analyses, it was found that males with age ≤ 55 and per capita family monthly income ≥ 2000 were more likely to execute the positive practice of undergoing pre-CRT (Table 5). This may be because as the age advances, patients are more likely to develop complications with preoperative treatment. Plus, low-income patients are equipped with less information and less guidance from their doctor; hence, they get to know less about the pre-CRT. These findings are consistent with some previous studies [18–21].

## 5. Conclusions

This study shows that, despite the fact that participants had suboptimal level of knowledge regarding rectal cancer, their attitude is favorable to pre-CRT. Inculcating into patients the knowledge of pre-CRT and enhancing their awareness of its importance may increase the uptake of preoperative therapy. In addition, our study suggests that age, gender, and income are significant factors contributing to patients' choice over pre-CRT.

## 6. Limitation and Expectation

Limited to the low proportion of implementation of preoperative chemoradiotherapy in advanced rectal cancer at present, we could not acquire a larger sample size regardless of our unremitting efforts. Therefore, it requires more subsequent trials to reach a more definitive conclusion. We are also looking forward to more relevant studies to confirm our conclusion.

## Figures and Tables

**Table 1:** Sociodemographic profile of participants and the association with practices (*n* = 111).

Characteristics	Number (%)	Univariate logistic regression analyses		
		OR^a^	95% CI^b^	*p* value
Age				
≤55	36 (32.4)	Reference		<0.001
56–64	28 (25.2)	0.134	0.043–0.415	<0.001
≥65	47 (42.3)	0.113	0.040–0.316	<0.001
Gender				
Female	37 (33.3)	Reference		
Male	74 (66.7)	3.279	1.412–7.615	0.006
Educational status				
Illiteracy	27 (24.3)	Reference		0.017
Primary	57 (51.4)	1.992	0.750–5.290	0.167
Secondary	22 (19.8)	6.333	1.814–22.107	0.004
High school and above	5 (4.5)	9.500	0.913–98.803	0.060
Occupation				
Unemployed	16 (14.4)	Reference		0.133
Employed	72 (64.9)	3.171	0.934–10.767	0.064
Retired	23 (20.7)	3.900	0.962–15.816	0.057
Marriage status				
Unmarried and other	11 (10.0)	Reference		
Married	100 (90.0)	2.041	0.577–7.218	0.268
Income (CNY)^c^				
≤1000	30 (27.0)	Reference		0.002
1001–1999	36 (32.4)	0.760	0.272–2.122	0.600
2000–2099	32 (28.8)	3.298	1.164–9.338	0.025
≥3000	13 (11.7)	9.500	1.771–50.957	0.009

^a^Odds radio; ^b^confidence interval.

^c^Chinese yuan (the currency of China).

**Table 2:** Knowledge, attitudes, and practices of rectal cancer pre-CRT.

Variables	*N* = 111 (%)	Pre-CRT (*n* = 54)	No Pre-CRT (*n* = 57)
Knowledge			
Know that CRT is treatment of rectal cancer	35 (31.5)	20 (37.0)	15 (26.3)
Know the importance of CRT	44 (39.6)	22 (40.7)	22 (38.6)
Know that pre-CRT can reduce local recurrence rate	76 (68.5)	47 (87.0)	28 (49.1)
Know that pre-CRT can reduce the side effects	76 (68.5)	47 (87.0)	28 (49.1)
Know that pre-CRT can keep anal sphincter function	76 (68.5)	47 (87.0)	28 (49.1)
Do not think pre-CRT will delay surgery timing	82 (73.9)	41 (75.9)	41 (71.9)
Attitudes			
Willing to go to a doctor	103 (92.8)	52 (96.3)	51 (89.5)
Do not worry about the side effects	65 (58.6)	31 (57.4)	34 (59.6)
Trust the doctor	106 (95.5)	50 (92.6)	56 (98.2)
Confidence in the treatment	66 (59.5)	37 (68.5)	29 (50.9)
Family's support and encouragement	97 (87.4)	52 (96.3)	45 (78.9)

**Table 3:** Approaches to get the knowledge of pre-CRT.

Approach	*N* = 111 (%)
Medical workers	44 (39.6)
Patient receiving radiotherapy	5 (4.5)
Television, newspapers, and other media	5 (4.5)
Network	5 (4.5)
Lack of knowledge	52 (46.9)

**Table 4:** Univariate logistic regression analyses about knowledge and attitudes with practices.

Variables (score)	*N* = 111 (%)	Practices (univariate analyses)		
		OR^a^	95% CI^b^	*p* value
Knowledge				
Low (0–2)	32 (28.8)	Reference		
Middle (3-4)	45 (40.5)	5.93	2.041–17.231	0.001
High (5-6)	34 (30.7)	7.944	2.560–24.657	<0.001
Attitudes				
Not favorable (0–3)	35 (31.5)	Reference		
Favorable (4-5)	76 (68.5)	3.437	1.450–8.150	0.005

^a^Odds radio; ^b^confidence interval.

^c^Chinese yuan (the currency of China).

**Table 5:** Multiple logistic regression analyses.

Variables	Multivariate analysis		
	OR^a^	95% CI^b^	*p* value
Age			
≤55	Reference		0.010
56–64	0.146	0.034–0.628	0.010
≥65	0.139	0.034–0.568	0.006
Gender			
Female	Reference		
Male	5.038	1.517–16.732	0.008
Income (CNY)^c^			
≤1000	Reference		0.013
1001–1999	0.978	0.245–3.908	0.974
2000–2099	7.034	1.543–32.077	0.012
≥3000	10.670	1.412–80.654	0.022
Knowledge			
Low	Reference		0.006
Middle	8.264	1.966–34.731	0.004
High	11.400	2.263–57.425	0.003
Attitudes			
Not favorable	Reference		
Favorable	8.522	2.297–31.616	0.001

^a^Odds radio; ^b^confidence interval.

^c^Chinese yuan (the currency of China).
